# Comparison of Tumor Cell Responses to Different Radiotherapy Techniques: Three-Dimensional Conformal Radiotherapy (3D-CRT), Volumetric Modulated Arc Therapy (VMAT), and Helical Tomotherapy (HT)

**DOI:** 10.3390/biology14050529

**Published:** 2025-05-10

**Authors:** Phanwadee Kasetthamrongrat, Rinwarat Phumsankhot, Aphidet Duangya, Anirut Watcharawipha, Wannapha Nobnop, Narongchai Autsavapromporn

**Affiliations:** 1 Medical Physics Program, Department of Radiology, Faculty of Medicine, Chiang Mai University, Chiang Mai 50200, Thailand; phanwadee_k@cmu.ac.th (P.K.); thanchanok_poom@cmu.ac.th (R.P.); 2 Division of Radiation Oncology, Department of Radiology, Faculty of Medicine, Chiang Mai University, Chiang Mai 50200, Thailand; aphidet.d@cmu.ac.th (A.D.); anirut.watch@cmu.ac.th (A.W.); wannapha.n@cmu.ac.th (W.N.)

**Keywords:** radiotherapy, three-dimensional conformal radiotherapy (3D-CRT), volumetric modulated arc therapy (VMAT), helical tomotherapy (HT), cancer cells, tumor response

## Abstract

Currently, advanced RT techniques such as VMAT and HT are being developed to optimize tumor coverage while minimizing radiation exposure to the surrounding organs that are at risk. Despite their growing clinical use, comparative studies evaluating the dosimetric and radiobiological effects of these modalities remain limited. In this study, A549, HeLa, and HepG2 cells were exposed to a single 2 Gy dose, using three RT techniques (3D-CRT, dual arc VMAT, and HT). Treatment plans were generated using a water phantom to ensure consistent target coverage and comparable dosimetric parameters across the techniques. Multiple radiobiological endpoints were assessed to evaluate the cellular responses. Although all three techniques yielded similar dosimetric parameters without statistically significant differences, the biological responses varied among the cell lines. Notably, VMAT and HT demonstrated superior tumor cell suppression compared to 3D-CRT. This was likely due to their enhanced dose conformity and modulation precision, which potentially led to improved tumor cell killing. These findings highlight the importance of integrating radiobiological assessments with physical dose metrics to inform the clinical application of advanced RT technologies. However, this study had several limitations. The use of a single radiation dose limited its clinical relevance, and the immediate post-irradiation assessments may not have captured delayed biological responses. Additionally, the small number of replicates may have reduced the study’s statistical power. Future studies incorporating dose fractionation schemes, time course analyses, and larger sample sizes are warranted to better simulate clinical conditions and further elucidate the radiobiological effects of advanced RT techniques.

## Simple Summary

Tumor responses to radiotherapy (RT) in cancer patients are influenced by a complex interplay of physical and radiobiological factors. This study aimed to evaluate the dosimetric parameters and biological effects on three human cancer cell lines after exposure to a 2 Gy radiation dose. We compared two advanced modulated radiotherapy techniques, Volumetric Modulated Arc Therapy (VMAT) and Helical Tomotherapy (HT), with the conventional non-modulated technique of Three-dimensional Conformal RT (3D-CRT). Although no statistically significant differences in the dosimetric parameters were found among the three techniques, variations in treatment planning factors—such as beam-on time, monitor units, and the radiation delivery system—appeared to influence the resulting biological outcomes. From a radiobiological perspective, tumor response is affected by DNA repair capacity, apoptosis, cell type, cell cycle distribution, and the bystander effect. Compared to 3D-CRT, both VMAT and HT demonstrated enhanced tumor cell killing, indicating the potential advantages of these advanced modulation techniques for improving RT efficacy.

## 1. Introduction

Cancer remains a major global health issue [1,2]. Radiotherapy (RT) is a crucial treatment modality that is used in over 50% of cancer patients, with the goal of maximizing the tumor dose while minimizing radiation exposure to the surrounding organs at risk (OARs) [3]. At our Cancer Treatment and Research Center, the available modern radiation technologies include Three-dimensional Conformal RT (3D-CRT), Intensity-modulated Radiotherapy (IMRT),Volumetric Modulated Arc Therapy (VMAT), and Helical Tomotherapy (HT) [3,4,5,6,7,8,9]. 3D-CRT utilizes three-dimensional imaging, such as computed tomography (CT) scans, to precisely shape radiation beams to match the tumor’s contour, enabling targeted radiation delivery while reducing the exposure of OARs [3]. IMRT, an advanced form of RT, delivers highly targeted radiation to tumors using computer-controlled multileaf collimators [4]. VMAT, a refinement of IMRT, delivers radiation in a continuous arc while dynamically adjusting the dose, beam shape, and gantry speed throughout treatment. This results in shorter treatment times and improved dose conformity [4,7]. HT integrates CT imaging and IMRT with a rotating linear accelerator, administering radiation in a helical pattern as the patient moves through the machine. This approach allows for precise dose modulation and the effective treatment of complex tumors while minimizing damage to OARs [5]. However, HT generally requires a longer delivery time compared to 3D-CRT, IMRT, and VMAT. VMAT is typically faster than IMRT due to its continuous dose delivery. Although both VMAT and HT require a higher number of monitor units (MU) than 3D-CRT and IMRT, the increased low-dose radiation exposure to surrounding OARs is primarily attributed to their rotational beam delivery [4,5,6,7,8,9,10].

Although delivering the same prescribed radiation dose, each radiation technique exhibits distinct physical and biological characteristics, potentially resulting in differences in clinical outcomes. Several studies have demonstrated that VMAT and HT significantly improve dose conformity and are better able to spare OARs compared to 3D-CRT and IMRT [11,12,13,14]. However, dosimetric comparisons between VMAT and HT have revealed only slight or no significant differences [15,16,17]. Currently, there is limited information regarding radiobiological responses that are directly compared to VMAT and HT modalities. Low-dose radiation delivered to surrounding OARs during VMAT and HT treatments may increase the risk of secondary cancers [6,10]. Additionally, the longer treatment times with VMAT and HT may reduce radiobiological effectiveness by allowing more time for sublethal damage repair in tumor cells [18,19]. To the best of our knowledge, this is the first study that has compared dosimetric parameters (D_95%,_ D_2%_, D_mean_, and homogeneity index [HI]) with biological responses among 3D-CRT, VMAT, and HT across three cancer cell lines (lung, cervix, and liver). The significance of these findings is discussed in relation to the dosimetric outcomes, the radiobiological effects across the three cancer cell lines, and their potential implications for the clinical application of these RT modalities.

## 2. Materials and Methods

### 2.1. Cell Cultures

In this study, we used three types of cell lines: human non-small cell lung adenocarcinoma (A549) cells, human cervical adenocarcinoma (HeLa) cells, and human hepatocellular carcinoma (HepG2) cells. All cell lines were obtained from the American Type Culture Collection (ATCC). The A549 and HepG2 cells were cultured in Dulbecco’s Modified Eagle Medium (DMEM), whereas HeLa cells were maintained in RPMI-1640 medium (Invitrogen, Thermo Fisher Scientific, Waltham, MA, USA). Both were supplemented with 10% fetal bovine serum, 100 U/mL penicillin, and 100 µg/mL streptomycin (Gibco, Life Technologies, Grand Island, NY, USA). Cells were cultured at 37 °C in a humidified incubator that contained 5% CO_2_.

### 2.2. Water Phantom Design

**Figure 1:**
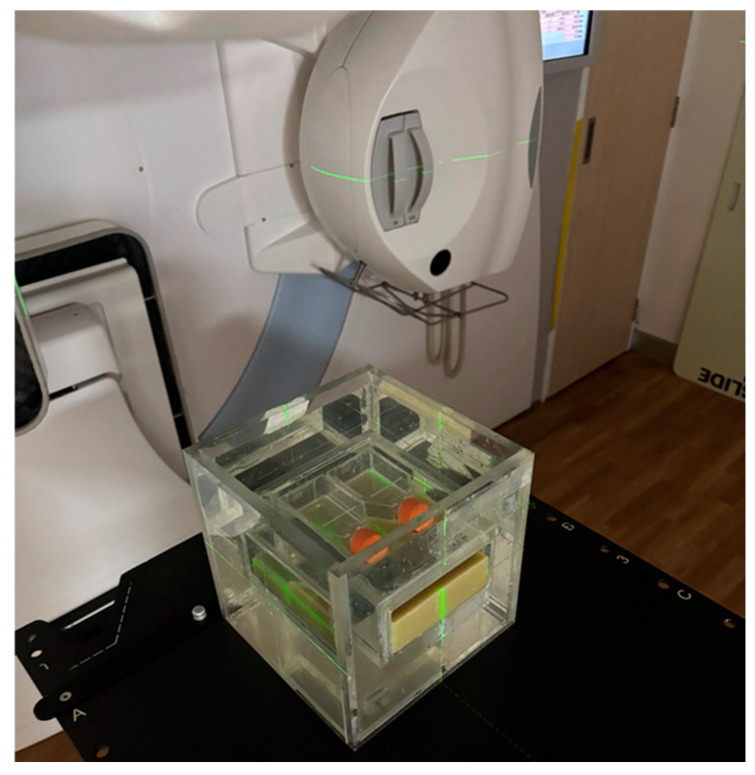
In the irradiation setup, cells were cultured in a T25 flask and positioned at the center of the water phantom.

Figure 1 illustrates the water phantom developed by our research team. The phantom had dimensions of 20.0 × 20.0 × 20.0 cm^3^, with an aperture measuring 13.0 × 16.0 × 5.0 cm^3^ at its centroid. For irradiation, a T25 flask (Corning^®^, Incorporated, Suzhou, China) was placed on a wax platform, which was specifically designed for this purpose, at the center of the phantom. The phantom was scanned with a slice thickness of 3 mm using a computed tomography simulator (SOMATOM Definition AS, Siemens Healthineers, Erlangen, Germany) for treatment planning. This image set was utilized for planning the 3D-CRT, VMAT, and HT treatments using a 6 MV photon beam.

### 2.3. Treatment Planning

**Figure 2:**
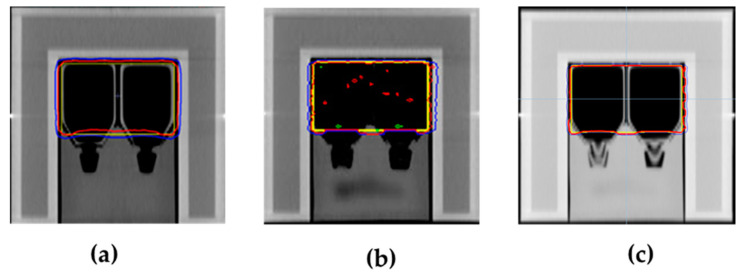
Isodose distributions with the planning target volume (PTV). (**a**) A 3D-CRT plan; (**b**) a dual arc VMAT plan; and (**c**) an HT plan. The yellow contour represents the PTV, the red line denotes the 100% isodose, the blue line indicates the 95% isodose, and the green line corresponds to the 2% isodose.

For the treatment planning, the structure of the entire flask was delineated and set as the target. The prescribed radiation dose for this target followed the recommendations of Nikolakopoulou et al. [20]. All treatment techniques were required to meet the criterion that at least 95% of the target volume should receive a radiation dose greater than 1.9 Gy (D_95%_ > 1.9 Gy). The mean dose was 2 Gy, while the maximum dose had to remain below 2.1 Gy. The homogeneity index (HI) was calculated according to the International Commission on Radiation Units and Measurements report, number 83 (ICRU No. 83), deriving as (D_2%_ − D_98%_)/D_50%_, where D_x%_ is the dose at x% target volume [21]. The beam geometry of each technique is illustrated in Figure 2 and described as follows: For the 3D-CRT plan, treatment planning was conducted with the Monaco^®^ treatment planning system (TPS) (Elekta, Stockholm, Sweden). The treatment technique utilized a four-field box arrangement, with beam angles set at 0°, 90°, 180°, and 270°. The radiation weighting was equally distributed at 25% for each beam direction. The dose calculation was performed using the collapsed cone convolution (CCC) algorithm. VMAT treatment planning was also performed using the Monaco^®^ TPS. A dual arc beam geometry, with a full 360° rotation, was utilized. To confine the radiation dose, a virtual structure (Ring structure) was incorporated to control the prescribed dose distribution. This treatment plan included 205 control points for modulated photon intensity. Dose calculation was performed using the Monte Carlo algorithm. The HT technique was planned using the Precision^®^ TPS (Accuray Incorporated, Sunnyvale, CA, USA). A virtual structure (Ring structure) was utilized for the same purpose as in the VMAT treatment technique. A rotational fan beam was set at 5.0 cm, using the dynamic jaws mode. The pitch factor and modulation factor were set to 0.287 and 2.3, respectively. Dose calculation was performed using the CCC algorithm.

### 2.4. Dose Verification

All treatment plans—3D-CRT, VMAT, and HT—underwent quality assurance to ensure accurate dose delivery to the water phantom. Patient-specific quality assurance (PSQA) was performed (point dose measurements) using a cubic water-equivalent phantom (Easy Cube, Lab Laser, Villetaneuse, France), equipped with a 0.01 cm^3^ ionization chamber (IC) (Scanditronix Wellhofer, Schwarzenbruck, Germany). Three ionization charge measurements were performed for each irradiation technique. IC readings were converted into absorbed dose values, which were then compared to the calculated doses. According to the criteria set by the American Association of Physicists in Medicine (AAPM), TG 119, the percentage difference between measured and calculated radiation doses should not exceed ± 3% [22]. Additionally, film dosimetry was performed for each cell irradiation session to ensure irradiation consistency. A radiochromic film (EBT3 film, Gafchromic, Ashland LLC, Bridgewater, NJ, USA) was placed beneath the T25 flask for dose measurement. The film was then analyzed for optical density (OD) and the corresponding radiation dose was calculated.

### 2.5. Irradiation Conditions

Irradiation was performed at the Division of Radiation Oncology, Department of Radiology, Faculty of Medicine, Chiang Mai University. All cell samples were cultured in T25 flasks and positioned at the center of a water phantom (Figure 1). For 3D-CRT and VMAT, the treatments were delivered using a 6 MV linear accelerator (Synergy, Elekta, Stockholm, Sweden), with dose rates of 600 MU/min and 1–600 MU/min, respectively. HT treatments were performed using a 6 MV Radixact X9 linear accelerator (Accuray Incorporated, Sunnyvale, CA, USA), with a dose rate of 1180 MU/min. The total beam on times for 3D-CRT, VMAT, and HT were 27.3, 125.8, and 103.2 s, respectively (Table 1).

### 2.6. Clonogenic Survival Assay

Following trypsinization and counting, the cells were seeded into P100 petri dishes and incubated for 9–14 days, depending on the cell type (A549—9 days; HeLa—12 days; and HepG2—14 days). After the incubation period, the culture medium was aspirated, and the cells were gently rinsed with phosphate-buffered saline (PBS) to remove the residual medium. Colonies were subsequently fixed using 99.5% ethanol, stained with a 0.5% crystal violet solution, and air-dried. Colonies consisting of more than 50 cells were identified and enumerated using light microscopy. The survival fraction (SF) was calculated based on the plating efficiency (PE) using Equations (1) and (2), as follows:

PE = Number of colonies formed/Number of cells seeded × 100%(1)

SF = PE of irradiated cells/PE of control cells(2)

### 2.7. Micronucleus Formation Assay

To evaluate the DNA damage in cancer cells, micronucleus (MN) formation was assessed using the cytokinesis-block technique [23]. In this experiment, ~5 × 10^4^ cells were carefully counted and seeded into 4-well chamber flasks, containing 2 µg/mL cytochalasin B (Sigma-Aldrich, St. Louis, MO, USA), prepared in D-MEM or RPMI-1640 culture medium. Incubation was performed for 48, 24, and 28 h for the A549, HeLa, and HepG2 cells, respectively, to facilitate binucleated cell formation. Upon the completion of incubation, the culture medium was aspirated and the cells were washed with PBS before fixation in 99.5% ethanol. Subsequently, the cells were stained with 1 µg/mL Hoechst 33342 solution and visualized under fluorescence microscopy. At least 1000 binucleated cells were scored per sample, with only micronuclei identified within the binucleated cells being considered for analysis.

### 2.8. Cell Cycle Assay

A cell cycle analysis was conducted using the Muse Cell Cycle Kit (MCH100106, Luminex Corporation, Austin, TX, USA) and a Muse^®^ Cell Analyzer (Merck Millipore, Burlington, MA, USA), following the manufacturer’s instructions. After irradiation, both the control and the irradiated cells (2 × 10^5^) were centrifuged at 300× *g* for 5 min, washed with PBS, and fixed in 70% ethanol at −20 °C for 3 h. Fixed cells (200 μL) were mixed with an equal volume of Muse^®^ Cell Cycle Reagent and incubated at room temperature, in the dark, for 30 min. The samples were then analyzed by flow cytometry, using the Muse^®^ Cell Analyzer system. The cell cycle data were presented as the percentage of cells in the G_0_/G_1_, S, and G_2/M_ phases.

### 2.9. Apoptosis Assay

An apoptosis assay was performed using the Muse Annexin V and Dead Cell Assay Kit (MCH100105, Luminex Corporation, Austin, TX, USA) and a Muse^®^ Cell Analyzer (Merck Millipore, Burlington, MA, USA), following the manufacturer’s instructions. After irradiation, both the control and irradiated cells (2 × 10^5^) were washed with PBS; mixed with 100 μL of Muse Annexin V and Dead Cell Reagent; and incubated for 20 min at room temperature, in the dark. Apoptotic cells were quantified by flow cytometry using the Muse^®^ Cell Analyzer system and the results were expressed as the percentage of apoptotic cells.

### 2.10. γ-H2AX Assay

The γ-H2AX assay was performed using the Muse™ H2A.X Activation Dual Detection Kit (MCH200101, Luminex Corporation, Austin, TX, USA) and a Muse^®^ Cell Analyzer (Merck Millipore, Burlington, MA, USA), following the manufacturer’s instructions. Following radiation exposure, both the control and irradiated cells (2 × 10^5^) were fixed and permeabilized before incubation with anti-phospho-histone H2A.X (Ser139) and anti-H2A.X antibodies. Subsequently, the cells were analyzed by flow cytometry, using the Muse^®^ Cell Analyzer system.

### 2.11. Statistical Analysis

All the results were expressed as the mean ± standard error (SE). A statistical analysis was performed using Sigma Plot version 10 (Systat Software, Inc., San Jose, CA, USA). Statistical analyses were performed on data obtained from at least two independent experiments, conducted on separate days. Poisson statistics and Pearson’s chi-square test were employed to compare independent samples. A *p* value of less than 0.05 was considered statistically significant.

## 3. Results

### 3.1. Dosimetric Results of Treatment Planning

Figure 2 shows the selected isodose distributions (100%, 95%, and 2%), along with the target for the 3D-CRT, dual arc VMAT, and HT plans. The dosimetric parameters, including D_95%_, D_2%_, D_mean_, and HI for the three plans are presented in Table 1. All plans revealed the average values for D_95%_, D_2%_, and D_mean_ as 2.00 ± 0.02, 2.09 ± 0.01, and 2.04 ± 0.02 Gy, respectively. Based on these findings, there was no significant difference in D_95%_, D_2%_, or D_mean_ among the three techniques. The HI of the treatment plans showed values of 0.08, 0.35, and 0.02 for 3D-CRT, dual arc VMAT, and HT, respectively. Among the three techniques, HT exhibited the lowest HI (0.02), suggesting the highest dose homogeneity, followed by 3D-CRT (0.08) and dual arc VMAT (0.35). Therefore, HT provided the most uniform dose distribution, making it the most effective technique in terms of dose homogeneity.

### 3.2. Verification Results of Dose Delivery

The results of the PSQA and dose consistency are presented in Table 2. All three plans showed a dose difference of 2.5% between the point dose measurements, with a 0.01 cm^3^ IC and the TPS dose calculation. All values were within the 3% tolerance recommended by AAPM TG 119 [22]. In the case of film dosimetry (EBT3 film), the dose consistency exhibited a variation of 3.10% when compared to the 3D-CRT plan. This indicated that the dose values obtained from all three techniques were comparable and within the acceptable range of variation, ensuring dosimetric consistency across different treatment methods.

### 3.3. Radiobiological Impact of Various Radiotherapy Techniques on Three Cancer Cell Lines

**Figure 3:**
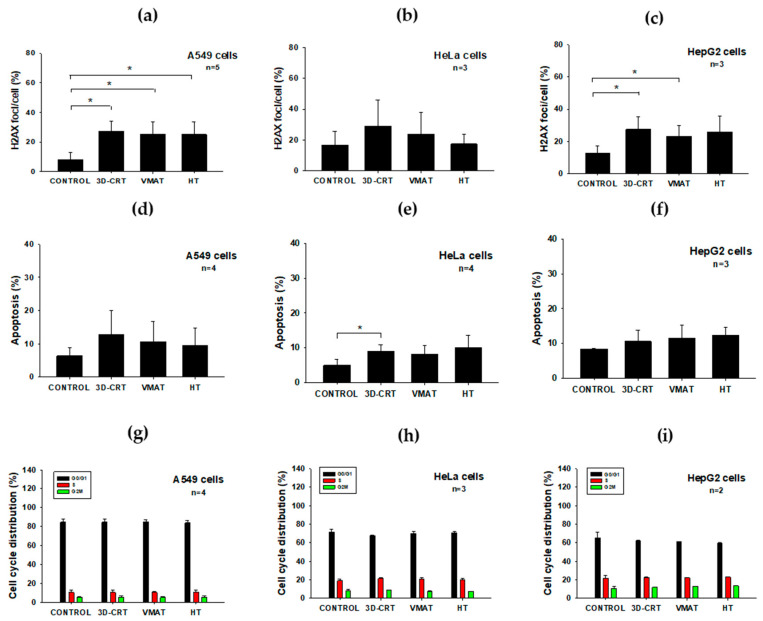
The biological effects of different radiotherapy techniques (3D-CRT, VMAT, and HT) on DNA damage (γ-H2AX foci), apoptosis, and cell cycle distribution in A549, HeLa, and HepG2 cell lines were analyzed immediately following irradiation with 2 Gy. (**a**–**c**) γ-H2AX foci per cell in A549 (**a**), HeLa (**b**), and HepG2 (**c**) cells indicating DNA damage. (**d**–**f**) Apoptosis rates in A549 (**d**), HeLa (**e**), and HepG2 (**f**) cells after irradiation. (**g**–**i**) Cell cycle distribution in A549 (**g**), HeLa (**h**), and HepG2 (**i**) cells; G0/G1 (black), S (red), G2/M (green). Data represent mean ± SD; *n* = 2–5; and * *p* < 0.05.

To evaluate the radiobiological impact of radiation exposure, the following biological endpoints were assessed immediately after irradiation. Figure 3a–c and Table 3 present the levels of γ-H2AX foci. This is an established marker of DNA double-strand breaks (DSBs), induced by ionizing radiation in A549, HeLa, and HepG2 cells following exposure to 2 Gy delivered by different RT techniques. In the A549 (Figure 3a) and HepG2 (Figure 3c) cells, exposure to 3D-CRT and VMAT resulted in a significant increase (*p* < 0.05) in γ-H2AX foci compared to the control, indicating the induction of DNA damage. In contrast, HeLa cells (Figure 3b) did not show a statistically significant difference among the irradiated groups and the control, suggesting a lower sensitivity to radiation or a more efficient DNA repair response in this cell type. As expected, when severe or irreparable DNA damage occurs—as indicated by the presence of γ-H2AX foci—cells may initiate apoptosis to prevent the propagation of damaged genetic material [24]. Figure 3d–f and Table 3 show the percentage of apoptotic cells post-irradiation. The HeLa cells (Figure 3e) displayed a statistically significant increase in apoptosis (*p* < 0.05) following 3D-CRT exposure compared to the control, suggesting that this technique may be more effective in inducing cell death. In contrast, the A549 cells (Figure 3d) and HepG2 cells (Figure 3f) exhibited no significant increase in apoptosis, indicating the potential resistance or delayed activation of apoptotic pathways following radiation exposure. These results indicated that the increase in γ-H2AX foci, a marker of DNA damage severity, does not consistently correlate with apoptosis across all cell types. This indicates that complex differences in DNA repair capability and apoptotic sensitivity accounted for the varied responses observed after exposure to different RT modalities. In this context, key DNA damage response pathways involving p53, ATM, and DNA-PK may modulate both γ-H2AX foci formation and apoptotic signaling, thereby shaping cellular radiosensitivity. These molecular mechanisms govern DNA DSB repair and cell fate decisions post-irradiation, and are likely to have contributed to the differential responses observed across cell lines and RT modalities [25].

To gain a deeper insight into the radiation sensitivity and cell-type-specific responses, we then analyzed the cell cycle distribution. Figure 3g–i and Table 3 illustrate the distribution of the cell cycle phases in the A549, HeLa, and HepG2 cells following radiation exposure. In all three cell lines, the proportions of irradiated cells in the G_0_/G_1_, S, and G_2_/_M_ phases remained relatively unchanged compared to the controls, indicating no clear evidence of radiation-induced cell cycle arrest after treatment with any of the three RT techniques. However, a subtle increase in the proportion of cells in the G_2_/_M_ phase was observed in the irradiated groups, particularly in the HeLa and HepG2 cells. This potentially reflected a modest G_2_/_M_ arrest—an established hallmark of the cellular radiation response [26]. These results suggest that the cell-type-specific responses to different RT techniques could guide the selection of treatment modalities in clinical settings.

**Figure 4:**
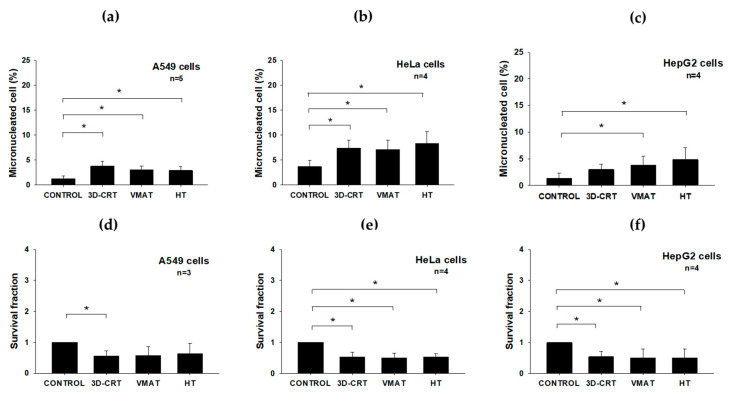
Micronucleus formation and survival fraction in A549, HeLa, and HepG2 cells assessed after exposure to 2 Gy of radiation delivered using different radiotherapy techniques: 3D-CRT, VMAT, and HT. (**a**–**c**) Percentage of micronucleated cells in A549 (**a**), HeLa (**b**), and HepG2 (**c**) cells following irradiation. (**d**–**f**) Survival fraction of A549 (**d**), HeLa (**e**), and HepG2 (**f**) cells assessed post-irradiation. Data represent mean ± SD from 3–5 independent experiments (* *p* < 0.05).

In addition to evaluating the immediate cellular responses through an analysis of the γ-H2AX foci and apoptosis, we further investigated the delayed radiobiological endpoints, specifically MN formation and SF, to comprehensively characterize the late effects of radiation exposure across different RT modalities. Figure 4a–f and Table 3 present the biological effects of radiation delivered via three different RT techniques—3D-CRT, VMAT, and HT—on MN formation and cell survival in A549, HeLa, and HepG2 cells. Unlike the immediate endpoints, such as the γ-H2AX foci and apoptosis, MN formation and SF reflect delayed or sustained biological responses to radiation exposure. MN formation (occurring 24–48 h after irradiation, depending on the cell type) is indicative of genomic instability and persistent chromosomal damage [23], which, in this study was significantly increased (*p* < 0.05) in the A549 cells following exposure to all three RT techniques (Figure 4a), suggesting radiation-induced chromosomal damage. In the HeLa cells, all three RT techniques led to a significant (*p* < 0.05) increase in MN frequency, indicating radiation-induced genotoxic effects across all modalities (Figure 4b). Similarly, the HepG2 cells showed significantly elevated MN formation (*p* < 0.05) following all RT treatments (Figure 4c), emphasizing sustained genomic instability induced by radiation.

Cell survival, which was assessed at 9–14 days post-irradiation (depending on the cell type), further underscored these delayed radiobiological effects. The A549 cells only exhibited significantly reduced SF (*p* < 0.05) after 3D-CRT compared to the controls. VMAT and HT showed non-significant reductions (Figure 4d, Table 3). These results indicate that cells are capable of repairing damage from MN formation caused by exposure to different radiation techniques (Figure 4a). In contrast, the HeLa cells exhibited significantly reduced survival fractions (SF) (*p* < 0.05) across all RT modalities, indicating pronounced radiosensitivity and inefficient DNA damage repair in this cell type (Figure 4b,e and Table 3). The HepG2 cells exhibited significantly reduced SF (*p* < 0.05) following all RT modalities, suggesting that this cell type displays marked radiosensitivity and deficient DNA damage repair mechanisms (Figure 4c,f and Table 3). Collectively, these findings have highlighted the distinct differences between immediate radiation responses, such as γ-H2AX formation and apoptosis, and late-effect outcomes, including MN formation and clonogenic survival. They emphasize the importance of evaluating sustained radiation effects, which are significantly influenced by DNA repair capacity, intrinsic radiosensitivity, and radiation delivery methods. These data support the rationale for cancer-patient-specific evaluation when selecting optimal RT modalities.

## 4. Discussion

**Figure 5:**
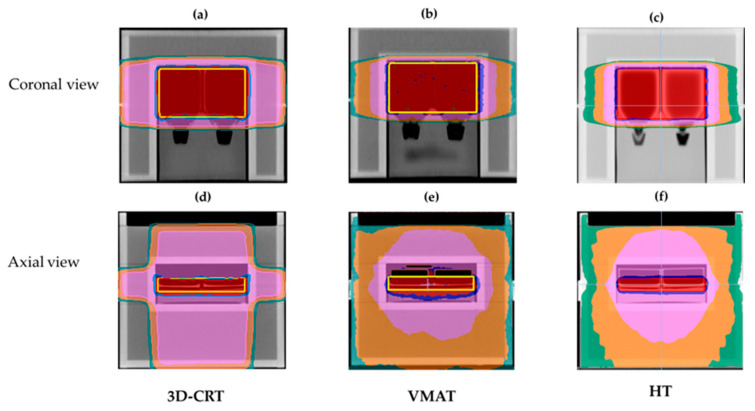
Treatment plans for 3D-CRT, VMAT, and HT, showing axial and coronal views of the water phantom. (**a**–**c**) Axial views of the dose distribution for 3D-CRT (**a**), VMAT (**b**), and HT (**c**). (**d**–**f**) Coronal views of the dose distribution for 3D-CRT (**d**), VMAT (**e**), and HT (**f**). Representative low-dose distributions are illustrated with isodose lines for 1 Gy (pink), 0.6 Gy (orange), and 0.4 Gy (dark green).

Advanced modulated techniques such as VMAT and HT offer superior dose conformity and homogeneity compared to 3D-CRT, enabling more precise tumor targeting and reduced radiation exposure to adjacent OARs [16,27]. Conversely, 3D-CRT generally results in less optimal dose conformity, often leading to higher radiation exposure to OARs [16,26]. Despite requiring longer planning and delivery times, VMAT and HT significantly enhance dose distribution and treatment outcomes, particularly in cases involving complex or irregularly shaped tumors [7,9]. Our results showed that, at a radiation dose of 2 Gy, there were no significant differences in the dosimetric parameters (such as D_95%_, D_2%_ and D_mean_) (Figure 2 and Table 1). However, the HI values obtained for HT and 3D-CRT were highly favorable, approaching zero, whereas the dual arc VMAT technique exhibited a higher HI value (0.35), indicating reduced dose homogeneity. This difference may be attributed to the dual arc rotational planning utilized in VMAT (Figure 2b, Table 1). Importantly, the MU and dose rates of VMAT and HT were substantially higher than those for 3D-CRT (Table 1). These differences may influence radiobiological responses, as higher dose rates can affect DNA damage repair kinetics, oxidative stress, and apoptotic signaling pathways. Such effects may contribute to low-dose hypersensitivity or modified cellular repair responses, even in the absence of large dosimetric differences [25,28]. Additionally, the VMAT and HT techniques resulted in a larger volume of tissue receiving low-dose radiation, potentially inducing a bystander effect that may inhibit tumor cell growth (Figure 5b,c,e,f), [5,7,29,30]. Therefore, investigating the radiobiological effects of these three techniques is essential, particularly given the lack of significant dosimetric differences. Such insights could help clinicians optimize treatment selection based on tumor type and patient-specific factors.

**Figure 6:**
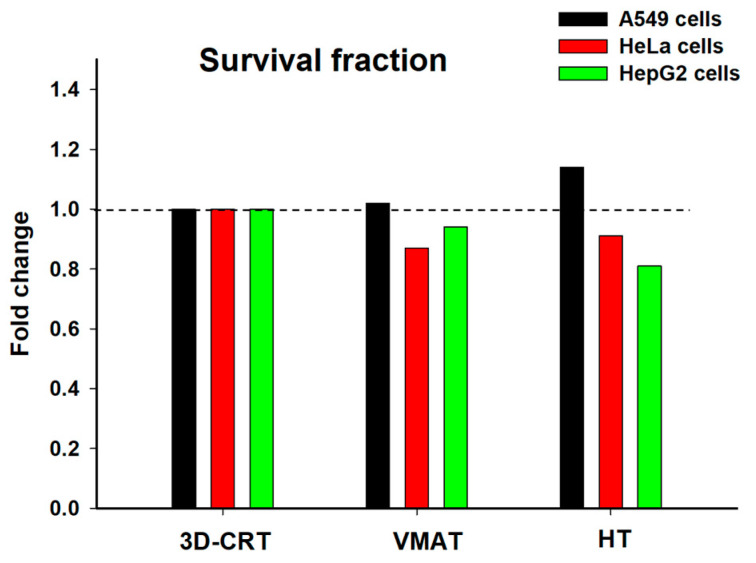
Fold change in the survival fraction of three human cancer cell lines—A549 (black), HeLa (red), and HepG2 (green)—after exposure to different radiotherapy techniques: 3D-CRT, VMAT, and HT. Data are normalized to the survival fraction observed with 3D-CRT for each respective cell line.

To compare the radiobiological impacts of different RT techniques, the SF of the A549, HeLa, and HepG2 cells were normalized to the 3D-CRT condition, which is the gold standard for evaluating cell survival in radiobiology (Figure 6), [31]. Under VMAT, the HeLa cells exhibited a noticeable decrease in SF, falling below 0.9, reflecting increased radiosensitivity compared to 3D-CRT. This finding suggests that VMAT may confer a more biologically effective dose to the cancer cells. The increased cell killing effect observed may, in part, be attributed to the high dose rate associated with VMAT, which has the potential to modulate cellular responses [7,28,32]. The HepG2 cells also demonstrated reduced survival, with an SF of approximately 0.9, indicating moderate radiosensitivity. In contrast, the A549 cells showed a minimal change in survival under VMAT, maintaining an SF close to 1.0, suggesting a response similar to that observed with 3D-CRT and indicating relative radioresistance under these conditions. This radioresistance may be partially explained by the intrinsic properties of A549 cells, including their predominant distribution in the G_0_/G_1_ phase of the cell cycle (~84%), which is relatively less sensitive to radiation compared to the G_2_/_M_ phase. The low proportion of cells in the more radiosensitive G_2_/_M_ phase (~5%), along with a reduced percentage in the S phase (~11%), where ongoing DNA replication can increase vulnerability to radiation-induced damage (Figure 3g), may have contributed to the limited killing effects observed following VMAT [26]. Furthermore, the A549 cells exhibited a lower frequency of radiation-induced MN compared to the other cancer cell lines (Figure 4a–c). The reduced MN formation suggests that A549 cells are more efficient at repairing DNA DSBs or are better at maintaining genomic stability by avoiding mitotic catastrophe [33]. These characteristics contribute to the development of an overall radioresistant phenotype.

In contrast, under HT, the A549 cells exhibited a slightly elevated SF (>1.0), further supporting their relative radioresistance. However, both the HeLa and HepG2 cells showed greater reductions in SF (approximately 0.8–0.85), indicating enhanced sensitivity to HT. This increased sensitivity may be attributed to their intrinsic biological characteristics and cell cycle distribution. Notably, both of these cell lines (Figure 3h,i) exhibited a higher proportion of cells in the G_2_/_M_ phase—approximately 12% for HepG2 and 8% for HeLa—which is considered the most radiosensitive phase due to chromatin condensation and limited DNA repair capacity [26]. This observation aligns with the elevated frequency of MN formation observed in both cell lines following HT exposure (Figure 4b,c), further supporting their heightened sensitivity to this technique. Together, these factors are likely to have contributed to the greater radiation-induced cell killing observed under HT in the HeLa and HepG2 cells. Additionally, HT’s unique delivery method—characterized by continuous helical irradiation, intensity modulation, and high dose conformity—may enhance the biological effectiveness of radiation by delivering a more uniform and precisely targeted dose over time. This approach may lead to greater DNA damage accumulation in rapidly dividing and less radioresistant cells, such as HeLa and HepG2, thereby reducing their survival more significantly than was the case with 3D-CRT or VMAT. Moreover, the observed reduction in SF may also be partially attributed to radiation-induced bystander effects (Figure 5). The low-dose regions inherent in HT delivery may also have triggered non-targeted responses, leading to cell death in the HeLa and HepG2 cells, whereas A549 cells appeared to be less affected. This suggests that the bystander effect may be cell-type specific, potentially due to differences in intercellular communication, signal transduction pathways, or DNA damage response mechanisms [34,35]. Overall, these results suggest that HeLa and HepG2 cells are more responsive to VMAT and HT than to 3D-CRT, with HT emerging as the most effective modality in significantly reducing the survival of these two cell lines. In contrast, A549 cells exhibited stable or slightly increased survival across treatment techniques, indicating a greater degree of radioresistance.

A major strength of this study lay in its novel comparison of both physical and biological effects across three radiotherapy techniques (3D-CRT, VMAT, and HT) on three distinct human cancer cell lines, using a water phantom to closely mimic human tissue. This experimental approach allowed for a more clinically relevant evaluation of radiobiological responses, providing potential guidance for clinicians in selecting the most appropriate RT technique based on tumor type. However, this study also had several limitations. First, the use of a single radiation dose limited our ability to fully understand the biological responses under realistic clinical conditions. Future research should incorporate fractionated dose regimens to better simulate clinical protocols and evaluate the repair and recovery capacity of cancer cells between fractions. Second, assessments and evaluations conducted immediately after irradiation may not fully capture the dynamic spectrum of biological changes. Future experiments should therefore include time course analyses to examine delayed or long-term cellular responses, facilitating a more comprehensive and accurate interpretation of radiation effects. Third, the lack of statistical significance in some comparisons may have reflected the study’s limited statistical power due to the small number of replicates (*n* = 2–5). Increasing sample sizes in future studies is recommended to strengthen the validity of the findings.

## 5. Conclusions

This study presented the first direct comparison of the physical and biological effects of three RT techniques (3D-CRT, VMAT, and HT), using three distinct cancer cell lines (A549, HeLa, and HepG2) within a clinically relevant water phantom model. The results demonstrated that VMAT and HT significantly enhanced radiosensitivity in HeLa and HepG2 cells compared to 3D-CRT, with HT showing the greatest reduction in cell survival. This enhanced efficacy is likely to have been due to HT’s high dose rate, continuous dose delivery, specific cell-cycle distributions, and the potential induction of radiation-induced bystander effects. Conversely, the A549 cells exhibited minimal or slightly increased survival following irradiation, indicating intrinsic radioresistance. This is likely to have correlated with their predominant distribution in the less radiosensitive G_0_/G_1_ phase and lower levels of DNA DSBs. While these findings have underscored HT’s potential as a superior modality for certain cancer types, this study had several limitations, including the use of a single radiation dose, immediate post-irradiation analyses, and the small number of replicates. Future research should incorporate fractionated irradiation schedules, extended post-irradiation time points, and increased numbers of biological replicates to more accurately capture the dynamic nature of radiobiological responses. Such approaches will be essential in optimizing treatment selection and improving the therapeutic outcomes tailored to specific tumor types. These findings have provided valuable insights into how different RT techniques may interact with specific tumor biology, reinforcing the potential for technique-specific radiosensitivity profiles. By integrating radiobiological endpoints into treatment planning, clinicians may be better equipped to personalize RT strategies based on tumor type, cellular response patterns, and inherent radiosensitivity. Such individualized approaches could improve treatment efficacy while minimizing toxicity, ultimately advancing the precision of clinical oncology. Overall, these results have highlighted the importance of integrating radiobiological endpoints with physical dose metrics to support the clinical implementation of advanced RT techniques.

## Figures and Tables

**Table 1:** Dosimetric parameters and irradiation conditions for the 3D-CRT, VMAT, and HT plans.

Parameter	3D-CRT	Dual arc VMAT	HT
D_95%_ (Gy)	1.98	2.01	2.00
D_2%_ (Gy)	2.10	2.08	2.08
D_mean_ (Gy)	2.05	2.02	2.05
HI	0.08	0.35	0.02
Beam on Time(s)	27.3	125.8	103.2
Monitor Unit (MU) rate	272.6	520.7	1782.8

**Table 2:** Comparison of treatment planning system (TPS) calculations with corresponding 0.01 cm^3^ ionization chamber (IC) point dose measurements for 3D-CRT, VMAT, and HT plans, along with a comparison of EBT3 film-measured dose with the 3D-CRT plan.

Technique	(%) Dose Difference	
	Point Dose Measurement (IC)	EBT3 Film
3D-CRT	−1.97	0
Dual arc VMAT	2.49	0
HT	0.67	3.1

**Table 3:** Comparative analysis of biological endpoints across different radiotherapy techniques in A549, HeLa, and HepG2 cells.

Cell Line	Technique	% γ-H2AX Foci/Cell	% Apoptosis	% Micronculeated Cell	Survival Fraction
A549	Control	9.37 ± 4.54	6.42 ± 2.34	1.28 ± 0.53	1.00 ± 0.00
	3D-CRT	26.88 ± 6.08	12.91 ± 7.16	3.76 ± 0.98	0.56 ± 0.17
	VMAT	24.37 ± 7.54	10.72 ± 6.03	3.00 ± 0.83	0.57 ± 0.30
	HT	23.94 ± 7.73	9.49 ± 5.30	2.94 ± 0.77	0.64 ± 0.33
HeLa	Control	16.86 ± 8.83	5.02 ± 1.70	3.68 ± 1.29	1.00 ± 0.00
	3D-CRT	28.91 ± 17.03	9.04 ± 1.82	7.38 ± 1.64	0.52 ± 0.16
	VMAT	23.95 ± 13.92	8.19 ± 2.45	7.10 ± 1.91	0.45 ± 0.11
	HT	17.42 ± 6.55	10.05 ± 3.57	8.28 ± 2.36	0.48 ± 0.04
HepG2	Control	11.23 ± 3.41	8.42 ± 0.14	1.40 ± 0.91	1.00 ± 0.00
	3D-CRT	24.92 ± 7.16	10.60 ± 3.18	3.00 ± 0.99	0.54 ± 0.17
	VMAT	20.17 ± 2.81	11.54 ± 3.83	3.85 ± 1.66	0.51 ± 0.28
	HT	23.76 ± 11.01	12.31 ± 2.44	4.85 ± 2.21	0.44 ± 0.18

## Data Availability

The data that support the findings of this study are available from the corresponding author upon reasonable request.

## Abbreviations

AAPM	American Association of Physicists in Medicine
CCC	Collapsed cone convolution
CT	Computed tomography
HI	Homogeneity index
HT	Helical tomotherapy
IC	Ionization chamber
ICRU	International Commission on Radiation Units and Measurements
MN	Micronucleus
MU	Monitor unit
OARs	Organs at risk
OD	Optical density
PBS	Phosphate buffer saline
PE	Plating efficiency
PSQA	Patient-specific quality assurance
PTV	Planning target volume
RT	Radiotherapy
SF	Survival fraction
TPS	Treatment planning system
VMAT	Volumetric modulated arc therapy
3D-CRT	Three-dimensional conformal radiotherapy
